# The Electronic Structures and Energies of the Lowest Excited States of the N_s_^0^, N_s_^+^, N_s_^−^ and N_s_-H Defects in Diamond

**DOI:** 10.3390/ma16051979

**Published:** 2023-02-28

**Authors:** Alexander Platonenko, William C. Mackrodt, Roberto Dovesi

**Affiliations:** 1 Institute of Solid State Physics, University of Latvia, 8 Kengaraga Street, LV-1063 Riga, Latvia; 2 Dipartimento di Chimica, Università di Torino, Via P. Giuria 5, 10125 Torino, Italy; 3 Accademia delle Scienze di Torino, Via Accademia delle Scienze 6, 10123 Torino, Italy

**Keywords:** N-doped diamond, C-centre, N_s_ defects, optical spectra, Δ-SCF calculations, Gaussian orbitals, B3LYP

## Abstract

This paper reports the energies and charge and spin distributions of the mono-substituted N defects, N^0^_s_, N^+^_s_, N^−^_s_ and N_s_-H in diamonds from direct Δ-SCF calculations based on Gaussian orbitals within the B3LYP function. These predict that (i) N_s_^0^, N_s_^+^ and N_s_^−^ all absorb in the region of the strong optical absorption at 270 nm (4.59 eV) reported by Khan et al., with the individual contributions dependent on the experimental conditions; (ii) N_s_-H, or some other impurity, is responsible for the weak optical peak at 360 nm (3.44 eV); and that N_s_^+^ is the source of the 520 nm (2.38 eV) absorption. All excitations below the absorption edge of the diamond host are predicted to be excitonic, with substantial re-distributions of charge and spin. The present calculations support the suggestion by Jones et al. that N_s_^+^ contributes to, and in the absence of N_s_^0^ is responsible for, the 4.59 eV optical absorption in N-doped diamonds. The semi-conductivity of the N-doped diamond is predicted to rise from a spin-flip thermal excitation of a CN hybrid orbital of the donor band resulting from multiple in-elastic phonon scattering. Calculations of the self-trapped exciton in the vicinity of N_s_^0^ indicate that it is essentially a local defect consisting of an N and four nn C atoms, and that beyond these the host lattice is essential a pristine diamond as predicted by Ferrari et al. from the calculated EPR hyperfine constants.

## 1. Introduction

The doping of diamond with nitrogen leads to a wide range of substitutional and vacancy-related defects that have been documented extensively (see reference [1] and references therein), including several computational studies of the ground state properties of mono-, di- and higher substitutional systems at various levels of sophistication. Of these, the neutral mono-substituted system, N_s_^0^, sometimes referred to as the C-center, has received more attention, with estimates [2,3] of the impurity and unpaired electron levels in the diamond gap and the related semi-conductivity [4], the local lattice structure [5,6], charge and spin distributions [5,6], infra-red and Raman spectra [7,8] and EPR hyperfine constants [5,6]. However, there would appear to be no detailed theoretical accounts of the optical properties, notably the strong absorption at 270 nm (4.59 eV) reported by Chrenko et al. [9], Walker [10], Nazare et al. [11] and more recently by Khan et al. [12] who also found weaker absorptions at 360 nm (3.44 eV) and 520 nm (2.38 eV), whose provenance is less certain than that at 270 nm. 

More recently, Jones et al. [13] pointed out that while the P1 signals and 270 nm optical absorption are both identified with the presence of N_s_^0^, and used widely to assess its concentration, the CVD grown diamond, for example, shows strong absorption at 270 nm apparently in the absence of, or at greatly reduced concentrations of N_s_^0^, at least as indicated by EPR [14]. At typical CVD temperatures of ~1100 K, which is more than twice the onset temperature of semi-conductivity [14], the concentration of N_s_^0^ would be expected to be extremely low resulting from the thermal excitation and subsequent dissociation of the resulting bound thermal exciton. This strongly suggests that the zero-spin defect N_s_^+^, which results from the ionisation of N_s_^0^, might also possess an optical absorption close to 270 nm [15]. It has also been argued [13] that since both natural and synthetic diamonds formed under a range of physical and chemical conditions exhibit optical absorption at 270 nm, N_s_^−^ and N_s_-H, which are both EPR inactive, might similarly contribute to the broad absorption in this region. 

Previous virtual spectra from several sources [1,2,3] have provided important information regarding the semi-conductivity in N_s_^0^, for they (i) predict a donor/defect band ~2 eV below the conduction band edge of the diamond host, which is clearly responsible for the semi-conductivity; (ii) show that the donor band comprises both N and C states [1,2,3]; and (iii) suggest that the semi-conductivity results from a phonon-driven, thermal transition. However, these spectra are limited in the detail they contain, particularly regarding the thermal transition and optical spectrum. For example, they do not reveal which components of the donor band are responsible for the thermal transition, nor which components of the valence and donor bands lead to the optical absorptions, nor the nature of the hybrid orbitals involved in both. Accordingly, this paper reports direct Δ-SCF calculations of the lowest energy excitations of N_s_^0^, N_s_^+^, N_s_^−^ and N_s_-H with three primary objectives. The first is to verify, or otherwise, the proposals by Jones et al. [13] concerning the defect states that contribute to the strong 270 nm optical absorption in the N-substituted diamond; the second is to give a detailed description of this and the weaker absorptions at 3.44 eV and 2.38 eV reported by Khan et al. [12]; and the third is to examine in more detail than has hitherto been available, the thermal transition responsible for the semi-conductivity ascribed to N_s_^0^ [4]. 

## 2. Materials and Methods

The calculations reported in this study are based on a direct Δ-SCF method, as implemented in the CRYSTAL code [16,17,18], and used previously to describe the lowest energy excitations in the largely ionic systems AF_II_ NiO [19] and α-Al_2_O_3_ [20], and more recently the highly local GR1 excitation of the neutral diamond vacancy [21] and the self-trapped exciton in diamonds [22], where the method has been described in detail in references [20] and [22]. The conceptual basis of the approach is that electronic excitation corresponds to the removal of an electron from the ground to a locally excited state that can be treated as a point defect in an otherwise perfect lattice using the long-established super-cell technique. Thus, Wannier–Mott excitons, for example, would be excluded from this approach. The essence of the computational procedure is that it calculates the ground and excited states separately, but identically, from which the difference in energy between the two states is obtained directly from the individual total energies. No restrictions are imposed on the excited state, other than the maintenance of zero occupancy of the donor orbital throughout the SCF procedure, and that electronic structures obtained in this way are fully relaxed to convergence of the total (excited state) energy, with computational conditions and accuracies that are identical to those of the ground state. In terms of the band structures, this corresponds exactly to the difference in energy between the ground and excited states at the Γ-point of the first Brillouin zone, and in terms of the optical spectra, the energy of the direct transition. For systems with indirect gaps, the excitation edge can be calculated from the excited state band structure, but this may not necessarily lie on one of the symmetry directions, and hence is not readily available to the same precision as the Γ-point energy. In principle, the use of a sufficiently large supercell for which the eigenvalues are folded back to the center of the first Brillouin zone (Γ) could also be examined. However, in the context of the present study, where the *identification* of the optical absorptions is the principal focus, accurate estimates of the excitation edge are not crucial. Since the excited state wavefunction is also obtained, the redistributions of charge and spin are straightforwardly estimated, while the full range of (excited state) dielectric and vibrational properties are also directly calculable.

The basis sets and computational conditions used in this paper were identical to those employed in a previous study of the ground-state vibrational properties and EPR constants of N_s_^0^ [3,23]. As before, the present calculations were based on the hybrid B3LYP functional [24,25] the general utility of which for estimating band gaps in crystalline materials was pointed out by Muscat et al. [26], while more recently, B3LYP has been shown to be superior to PBE0 [27], HSE06 [28] and GGA [29] in direct Δ-SCF studies of low-lying excited states in AF_II_ NiO [19] and the self-trapped exciton in diamond [22]. Modified Pople 6–21G basis sets, {(1s)(2s,2p_x_,2p_y_,2p_z_)(3s,3p_x_,3p_y_,3p_z_)}, were used for C and N, with the exponents of the outer-most sp orbitals set to 0.23 Bohr^−2^ and 0.30 Bohr^−2^, respectively, and the truncation of the Coulomb and exchange infinite series controlled by five thresholds T_i_ [16], which were set to 8 for (T_1_–T_4_) and 16 for T_5_. The convergence threshold on energy for the self-consistent-field (SCF) procedure was set to 10^−8^ E_h_ for structural optimizations and to 10^−11^ E_h_ for the frequency calculations. Supercells containing 64 atoms, and, where necessary, 128 atoms, were used to simulate the defective system, with a shrinking factor of 8 leading to 105 k-points in the IBZ (irreducible Brillouin zone). 

As in previous studies of excited states in diamond [21,22], where the (covalent) bonding is described in terms of suitable sp^3^ hybrid orbitals that are used directly in the Δ-SCF procedure to calculate 1-electron excited state energies and wavefunctions, here, similar C, N and CN hybrid orbitals were constructed from combinations of atomic orbitals such that the sum of the ground state orbital populations, i.e., the total hybrid populations, were ~1ǀeǀ and that the local symmetry was preserved. Net atomic charges and bond populations between atoms were obtained from the widely used Mulliken partition of the total charge density (and similarly for the corresponding spin quantities), as discussed in detail by Pascale et al. [30].

## 3. Results

### 3.1. Ground State

**Figure 1:**
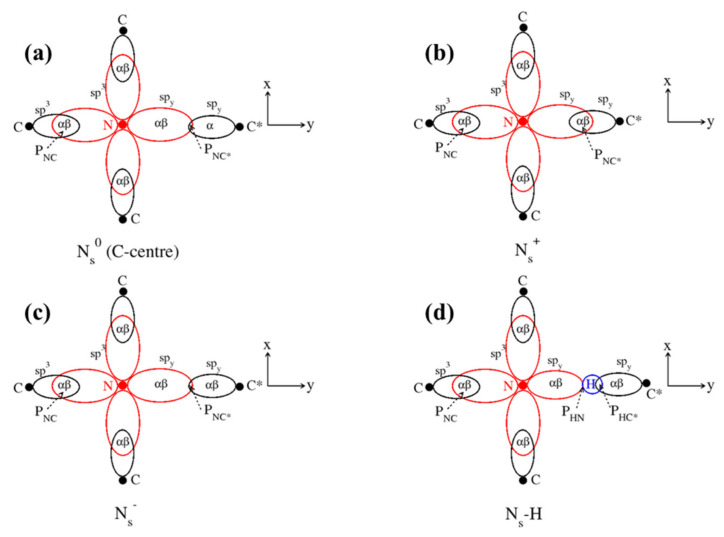
Two-dimensional stereographic projections of all four systems: (**a**) N^0^_s_, (**b**) N^+^_s_, (**c**) N^−^_s_, (**d**) N_s_-H. P_NC/NH_ indicates bond populations.

We begin with the ground states both for the information they contain and the essential role they play in the Δ-SCF approach to excited states used in this paper. The optimized ground state structures of N_s_^0^, N_s_^−^ and N_s_-H are locally trigonal about an axis along N and the electron-deficient C atom in N_s_^0^, C*, whereas N_s_^+^ retains the tetragonal symmetry of the host lattice. Two-dimensional stereographic projections of all four systems are shown in Figure 1, where N-C* is along the y-axis in each case. 

Table 1 lists the optimised nearest neighbour (nn) N-C distances (Å), r_N-C*_, r_N-C_, and B3LYP/6-21G atomic charges (ǀeǀ), q_N_, q_C*_, q_C_, atomic spins (ǀeǀ), s_N_, s_C*_, s_C_, and overlap charges (ǀeǀ), P_NC*_, P_NC_, P_HN_, P_HC*_ in N_s_^0^, N_s_^+^, N_s_^−^ and N_s_-H. The spin of the un-paired electron in Ns0, which is formally located at C*, is predicted to delocalise at N, leading to hyperfine spin-spin interactions with the N nuclear spin, as observed previously [5,6]. In view of the role played by the un-paired electron in the optical spectra, we note that the B3LYP/6-21G prescription used in the present study predicts the Fermi contact term within 2% and the anisotropic dipole-dipole components of the hyperfine coupling tensor to within 11% of the measured values [3]. In all four systems, q_N_ is ~−0.5 ǀeǀ, whereas q_C*_ varies from 0.14 ǀeǀ in N_s_^0^ and N_s_^+^ to −0.12 ǀeǀ in N_s_-H. The bond populations, P_x_, are instructive for they confirm the expected bonding between N and C in all four cases (P_NC_ ~0.3 ǀeǀ) and between N and C* in N_s_^+^ (P_NC*_ 0.2 ǀeǀ), with a net anti-bonding N-C* interaction predicted in the three trigonal systems (P_NC*_ < 0). As expected, the present calculations predict a strong covalent bond between H and C* orbitals (P_HC*_ 0.3 ǀeǀ), with H and N non-bonded.

**Figure 2:**
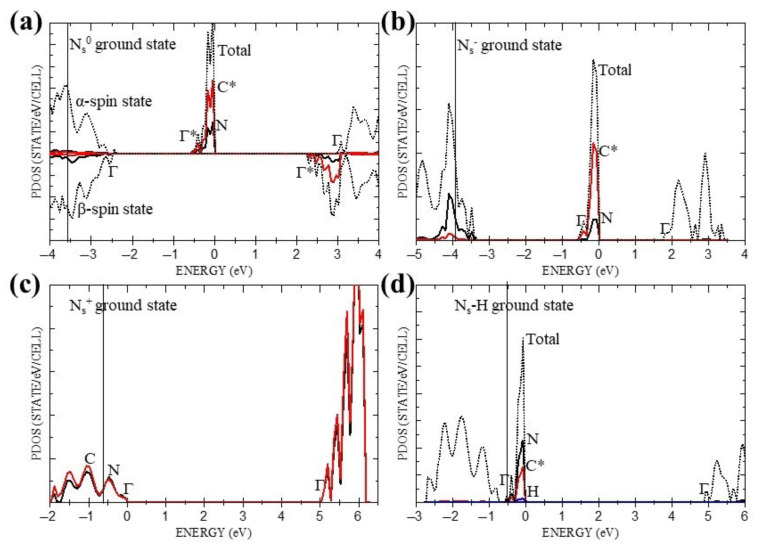
Ground states PDOS for all four systems: (**a**) N_s_^0^, (**b**) N_s_^−^, (**c**) N_s_^+^, (**d**) N_s_-H. Red solid, black solid, blue solid and black dotted lines correspond to carbon*, nitrogen, hydrogen and total DOS, respectively. C* and N refer to Figure 1 and Γ indicates the Γ- points of the individual bands. Vertical solid line indicates a diamond (host) valence band edge.

We turn now to band structures, for they have been the principal theoretical means for explaining the donor and acceptor levels and related semi-conductivity in N_s_^0^, and providing estimates of the optical spectra [1,2,3]. Figure 2a–d show the ground-state partial densities of states (PDOS) of N_s_^0^, N_s_^+^, N_s_^−^ and N_s_-H, where the diamond (host) valence band edge is indicated by a solid vertical line and measured from the local virtual band in each case. For N_s_^0^, the α-band is taken as the majority spin. These indicate that the local states in N_s_^+^ are only minimally changed from the host value, whereas N_s_^0^, N_s_^−^ and N_s_-H are all predicted to contain a local (filled) defect band, ~0.6 eV wide, derived from both N and C* states. However, only N_s_^0^ and N_s_^−^ with indirect gaps of ~2.2 eV and ~1.8 eV, are compatible with the reported activation energy for semi-conductivity of ~1.7 eV [4]. 

For completeness, and as a measure of the reliability of the computational methodology used in this study, Table 2 lists the previously reported [19] band gap energies of the host lattice, where Eg is the calculated un-normalised indirect band gap, E_Γ_ the lowest direct gap (Γ_25′_-Γ_15_), ZPE the zero point energy and nEg the normalised gap, (Eg-ZPE), which was introduced by Cardona et al. [31] to interpret the temperature dependence of the experimental exciton energy gap.

Table 3 lists the calculated spin-allowed (Δs_z_ = 0) and spin-forbidden (Δs_z_ ǂ 0) band gaps (virtual transition energies) for N_s_^0^, where the indirect and direct α → α transitions are potential candidates for the weak absorptions at 2.38 eV and 3.44 eV, respectively, and the direct (Γ- Γ) β → β transition a candidate for the strong 4.59 eV optical absorption, while the spin-forbidden indirect α → β transition can be identified with the activation energy for the thermal (phonon-mediated) semi-conductivity. 

Table 4 lists the predicted gaps in N_s_^+^, N_s_^−^ and N_s_-H, from which E_g_ in N_s_^−^ is in the vicinity of the thermal activation energy of ~1.7 eV, and two indirect transitions in N_s_-H possible contributors to the strong optical transition.

### 3.2. Excited States

Starting with N_s_^0^, Figure 2a indicates that there are three bands from which low-energy excitations originate. They are the α-spin donor band which, strictly speaking, is part of the α valence band but for clarity is considered separately from the remainder of the band, which we refer to as the α valence band, and then the other part is the β valence band. In addition to the two criteria referred to in Section 2 which determine the choice of suitable hybrid orbitals to be used directly in the Δ-SCF procedure, in the case of the donor band there is further restriction, namely, that the PDOS shown in Figure 2a requires the ratio of the C* to N orbital densities in the ground state to be ~3. This restricts the choice to a single hybrid namely, {N(2s) + C*(2p_y_) + C*(3p_y_)} which we write more simply as N(2s)C*(2p_y_,3p_y_), as shown in Table 5, together with the predicted Γ-point energy, Δ_SCF_, absorption edge, E_g_, and changes in atomic charges, δq_N_, δq_C*_, δq_C_ and spins δs_N_, δs_C*_, δs_C_, resulting from the transition. In the context of the present study, which is concerned with the absorption spectra, the term ‘absorption edge’ is preferred to ‘band gap’ for excited states. Unlike the diamond host, where the dispersion of the lowest unoccupied band is ~1.6 eV between the Γ-point (Γ_15_) and the minimum energy close to Δ1, the corresponding bands in N_s_^0^, N_s_^+,^ N_s_^−^ and N_s_-H are appreciably flatter, with dispersion energies ranging from 0.04 eV for N_s_^0^ to 0.16 eV N_s_^−^. Thus, for a good approximation, indirect absorption edges, E_g_, are given by the directly calculated energy (ΔSCF) minus the band width, with no ZPE corrections. The full list of directly calculated hybrid excitation energies below the strong absorption edge of the diamond host (~5.5 eV), together with the corresponding changes in atomic charge and spin at the N and nearest neighbour C sites, are collected in Table 5. Similarly, the energies of the possible hybrid excitations and changes in local charge and spin for N_s_^+^, N_s_^−^ and N_s_-H are collected in Table 6, Table 7 and Table 8.

## 4. Discussion

The optimised local ground state structure of N_s_^0^ is predicted to have C_3v_ symmetry about the N-C* direction, in agreement with deductions from the EPR data and previous calculations [3]. N_s_^−^ and N_s_-H, which have one more electron but no net spin, are also predicted to possess a similar local trigonal symmetry, while N_s_^+^ with eight valence electrons associated with the inner quintet of atoms, has local T_d_ symmetry, as expected. As in previous studies [2,3], the ground state PDOS, shown in Figure 2a–d, and related virtual spectra provide a useful initial assessment of the possible thermal and optical excitations. Thus, as Table 3 indicates, the direct α → α and β → β excitations of N_s_^0^ with energies of 3.62 eV and 4.60 eV, respectively, are seen to be consistent with the reported weak and strong optical absorptions at 3.44 eV and 4.59 eV [12]; while the direct and indirect α → β energies of 2.75 eV and 2.22 eV are also consistent with the weak optical absorption at 2.38 eV [12], and previously reported peak at ~2.3 eV in the photo-conduction spectra [4], and the activation energy for semi-conductivity of ~1.7 eV [4]. On the other hand, the virtual spectra of N_s_^+^, N_s_^−^ and N_s_-H shown in Table 4 suggest negligible contributions to the reported optical absorption from these systems, apart from N_s_^−^ which may participate in the weak optical absorption at 2.38 eV [12] and the semi-conductivity in N-doped diamonds. However, as useful as it is, this description is incomplete, for it lacks specific details of the atoms and orbitals involved in the excitations cited.

Turning now to direct Δ-SCF calculations, and starting with the donor band in N_s_^0^, these find the only excitation below the strong absorption edge of the diamond host to be an α → β {N(2s)C*(2p_y_,3p_y_)} excitation with a Γ-point energy, Δ_SCF_, of 1.96 eV, as reported in Table 5. This is 0.79 eV lower than that obtained from the virtual spectrum. With reference to Figure 2a, the associated absorption edge, E_g_, corresponds to an excitation from the donor band maximum leading to a value of 1.43 eV, with the absorption peak in the interval (1.43–1.96) eV, which follows from the zero densities of states at donor band extrema. Since this is a spin-flip excitation (ΔS_z_ ǂ 0), the optical intensity is expected to be extremely low as the observed absorption spectrum indicates [12]. Regarding the ‘activation energy’ for semi-conductivity in N_s_^0^, which is widely attributed to the thermal excitation of the donor band, it is important to recognize that this is an average energy for conduction over a range of temperatures, with an onset at ~500 K (~0.04 eV) [4]. As such, it results from multiple inelastic phonon scattering events with a net energy transfer that is approximately the energy of the thermally excited state of the {N(2s)C*(2p_y_,3p_y_)} hybrid, which is written in Table 5 as {N(2s)C*(2p_y_,3p_y_)}t. Thus, the weak absorption at 2.38 eV [12] might be associated with the optical excitation of a {N(2s)C*(2p_y_,3p_y_)} hybrid, and the ‘activation energy’ for semi-conductivity to the corresponding thermal excitation. Mulliken analysis of the optical excited state wavefunction indicates that the transition involves a *net* transfer of charge (δq_N_) of ~0.3 (ǀeǀ) from N to C* and the three nn C atoms together with a re-distribution of spin (δs) resulting from a back donation of charge. The italicised values in Table 5, which correspond to the differences between the optically and thermally excited states, show that further re-distributions of charge and spin in the thermally excited state are negligible.

Δ-SCF calculations find two possible hybrid excitations from the valence bands in N_s_^0^ to which the optical absorptions at 4.59 eV and 3.44 eV [12] can be attributed. They are the β→β and α→β N(3s,2p_y_) and N(3s,3p_y_) excitations, with Γ-point energies of 4.72 eV and 3.04 eV respectively. Furthermore, the α→α N(3s,3p_y_) excitation, for which the absorption edge and Γ-point energies are 3.67 eV and 4.23 eV respectively, cannot be discounted from contributing to the strong absorption at 4.59 eV, for the width of the observed absorption reported by Khan et al. [12] is ~0.5 eV, while both Nazare and Neves [11] and Tallaire et al. [14] have observed absorption from ~4.1 eV to ~4.7 eV in this region as noted previously by Jones et al. [13]. Mulliken analyses of the excited state wavefunctions indicate that all five excitations lead to a net charge transfer from N to the four nn C atoms, including C*, ranging from −0.55 (ǀeǀ) to −0.80 (ǀeǀ) for the N(3s,2p_y_) and N(3s,3p_y_) excitations, respectively. Again, there is an appreciable re-distribution of spin resulting from the charge-compensating back donation of charge to the N. There are also two possible α → β C* hybrid excitations, namely, C*(3s,2p_y_) and C*(2s,2p_y_,3p_y_) which have not been reported, and in any event will be weak. The first is an excitation from C* to its three nn C atoms, which is not shown in Table 5; the second is a pure spin flip with no charge transfer, but simply a re-distribution of spin.

In contrast to the ground state virtual spectrum of N_s_^+^ shown in Figure 2b which contains a direct absorption edge close to 5 eV, Δ-SCF calculations predict several N and C* hybrid excitations which are listed in Table 6, but which carry an important caveat. It is the assumption that the uniform negative field used to stabilize the Madelung potential, leads to identical shifts in the total energies of the ground and excited states, despite the re-distribution of charge in the excited state. While this is a reasonable assumption, it remains without proof. The hybrid criteria outlined previously lead to two possible N excitations below the host absorption edge. They are N(3s,2p_y_) and N(3s,3p_y_), with direct energies of 5.16 eV and 2.34 eV, respectively, which compare with values of 4.23/4.72 eV and 3.04 eV for the corresponding excitations in N_s_^0^. In addition, there are three possible C* excitations, C*(2s,3s,2p_y_,3p_y_), C*(2s,3s,2p_y_) and C*(3s,2p_y_,3p_y_), with energies of 4.66 eV, 4.17 eV and 2.51 eV, respectively. Mulliken analyses of the excited state wavefunctions collected in Table 6, indicate that the two N excitations lead to charge transfers to its four equivalent nn C atoms (including C*), whereas the C* excitations lead to charge transfers to both N and its three nn C atoms. For all five excitations, there are appreciable re-distributions of spin resulting from back donations of charge. The C*(2s,3s,2p_y_,3p_y_) and C*(2s,3s,2p_y_) are particularly significant for they support the proposal by Jones et al. [13] that N_s_^+^ is the likely source of the ~4.6 eV optical absorption in circumstances when the concentration of N_s_^0^ is either extremely low, or absent altogether [14,15]. Furthermore, the present calculations predict that the weak absorption at ~2.4 eV in similar circumstances can also be attributed, in part, to N_s_^+^.

The ground state PDOS of N_s_^−^ shown Figure 2c suggests a single low energy excitation with an absorption edge at 1.81 eV and Γ-point energy at 2.55 eV, which can plausibly be attributed to the weak ~2.4 eV peak in the optical [12] spectrum. As in the case of both N_s_^0^ and N_s_^+^, Δ-SCF calculations listed in Table 7 posit a greater number of possible excitations, with four below the diamond edge, of which two are predicted to lead to conducting states. Of the remaining two, the N(3s,3p_y_) excitation could contribute to the weak 3.44 eV optical peak [12], at least on energy grounds, for the absorption peak is expected to lie in the interval 3.36 eV–3.94 eV. Similarly, the C*(3s,2p_y_,3p_y_) excitation might contribute to the optical [12] peaks at ~2.4 eV. Both excitations involve strong charge transfer, with net depletions of 0.77 (ǀeǀ) and 0.68 (ǀeǀ) to their respective nn C atoms. However, the caveat regarding the effect of the uniform electric field once again applies. It is important to emphasise that the calculations reported here for N_s_^+^ and N_s_^−^ cannot predict the presence of these defects in N-substituted diamond, but simply their excitation energies and possible contributions to the optical spectrum if there are grounds for inferring their presence [12,13].

Jones et al. [13] and Khan et al. [12] have surmised that under suitable conditions, N-substituted diamond might contain N_s_-H defects, which could make a contribution to the optical spectra. For completeness, therefore, we have examined their low-energy excitations in relation to the reported optical spectrum of N-substituted diamond. B3LYP calculations find N_s_-H to be a wide band gap insulator, as shown in Figure 2d, where the direct and indirect gaps are listed in Table 4. This contains two indirect gaps at 4.45 eV and 4.63 eV, which could reasonably be associated with the ~4.6 eV optical absorption [12]. Δ-SCF calculations given in Table 8 suggest otherwise, with a predicted peak between 3.75 eV and 4.32 eV for the sole excitation, C*(2p_y_,3s,3p_y_), below the diamond edge. As Table 8 indicates, this involves a transfer of charge from C* to H and its three nn C atoms.

**Figure 3:**
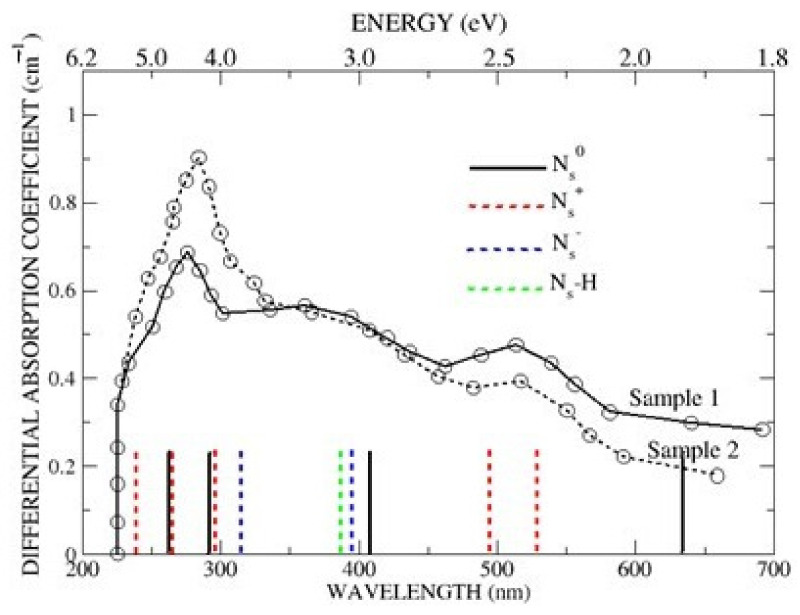
Comparison of the predicted Δ-SCF Γ-point excitation energies in N_s_^0^ (black), N_s_^+^ (red), N_s_^−^ (blue) and N_s_-H (green) with the optical spectra reported in Figure 4, Khan et al. [12]. (The heights of the vertical lines are arbitrary).

A clearer perspective of the Δ-SCF energies is shown in Figure 3, where the Γ-point energies for N_s_^0^ (black), N_s_^+^ (red), N_s_^−^ (blue) and N_s_-H (green) are compared with the optical spectra reported by Khan et al. [12]. For clarity, the hybrid designations of the individual Γ-point energies are not included but are readily found in the text. Three significant points emerge from Figure 3. The first is that N_s_^0^ and its two singly ionised states, N_s_^+^ and N_s_^−^ are predicted to absorb in the region of 4.6 eV, with the exact contributions depending on the experimental conditions. The spectra shown in Figure 3 were obtained at ~800 K [12], well above the onset temperature of ~450 K for semi-conductivity [4], in which case the samples would reasonably be expected to contain both N_s_^0^ (black) and N_s_^+^ (red). Under conditions which favour the formation of N_s_^−^ (blue), calculations suggest that it, too, would contribute to the strong absorption. Second, Figure 3 shows that the very weak absorption at ~400 nm, notably in the Sample 2 spectrum, might be attributed to N_s_^0^ (black), N_s_^−^ (blue) and N_s_-H (green), although the predicted absence of N_s_^+^ (red) suggests N_s_-H, or some other impurity, as the likely candidate, with the intensities of the N_s_^0^ and N_s_^−^ excitations too weak for detection. Third, the optical absorption at ~500 nm (~2.4 eV) is attributed to the {N3s,3p_y_)} and C*(3s,2p_y_,3p_y_)} excitations of N_s_^+^ for exactly the same reason it contributes to the 270 nm (~4.6 eV) absorption, with the α → β {N(2s)C*(2p_y_,3p_y_)} excitation of the donor band again too weak for detection.

Finally, we consider briefly a generic issue related to point defects in solids generally, but here, related specifically to N_s_^0^. It concerns the extent of the perturbation to the host lattice. The observed EPR hyperfine coupling constants and previous B3LYP calculations by Ferrari et al. [3] find values of A_iso_ and B_1,2,3_ for C atoms beyond nearest neighbour (nn) to N to be 5%, and less, than those at C*, which suggests that N_s_^0^ can properly be described as a local defect consisting of N and four nn C atoms. Δ-SCF calculations of the exciton energy (ΔE_SCF_) confirm this with values of 7.33 eV for the nn C atoms (Shell 1), 7.22 eV for C atoms once removed (Shell 2) and 7.24 eV, which is the bulk value, thereafter. Thus, for closed shell systems, the calculated exciton energy at different atomic sites from a defect might provide a useful measure of its spatial extent.

## 5. Conclusions

The principal conclusion of this study is that it confirms previous reports that the direct Δ-SCF approach to excited states provides information about excited states that is not readily available by other means. More specific conclusions are that,

- The semi-conductivity in N-doped diamond, with an onset at ~500 K (~0.04 eV) [4] and activation energy of ~1.7 eV [4], results from multiple inelastic phonon scattering events with a net energy transfer that is approximately the energy of the thermally excited state of the {N(2s)C*(2p_y_,3p_y_)} hybrid of the donor band.
- N_s_^0^ and its first two ionised states, N_s_^+^ and N_s_^−^ all absorb optically in the region of 4.6 eV (270 nm), with the contributions from the ionised states dependent on the experimental conditions. Thus, direct Δ-SCF calculations support the important suggestion by Jones et al. that N_s_^+^ contributes to, and in the absence of N_s_^0^ is responsible for, the 4.59 eV optical absorption in N-doped diamond.
- N_s_-H or some other impurity is responsible for the weak optical absorption reported by Khan et al. [12].
- N_s_^+^ is the source of the weak absorption at ~2.4 eV (520 nm) for the same reason it contributes to the 4.6 eV (270 nm) absorption.
- The predicted energies of the self-trapped exciton close to N_s_^0^ confirm previous calculations of Ferrari et al. [3] that it is essentially a local defect consisting of a N and four nn C atoms and that beyond these the host lattice is an essential pristine diamond.

## Figures and Tables

**Table 1:** Optimised (nn) N-C distances (Å), r_N-C*_, r_N-C_ and B3LYP atomic charges (ǀeǀ), q_N_, q_C*_, q_C_, atomic spins (ǀeǀ), s_N_, s_C*_, s_C_, and overlap charges (ǀeǀ), P_NC*_, P_NC_, P_HN_, P_HC*_ in N_s_^0^, N_s_^+^, N_s_^−^ and N_s_-H. Numbers in brackets are the % difference from the diamond C-C distance.

	N_s_^0^	N_s_^+^	N_s_^−^	N_s_-H
r_N-C*_	2.05 (+27%)	1.57 (+0.8%)	2.26 (37%)	2.36 (40%)
r_N-C_	1.50 (−4%)	1.57 (+0.8%)	1.47 (−6%)	1.48 (−5%)
q_N_	−0.42	−0.42	−0.44	−0.56
q_C*_	+0.14	+0.14	−0.05	−0.12
q_C_	+0.09	+0.14	+0.15	+0.15
s_N_	0.20 α	-	-	-
s_C*_	0.76 α	-	-	-
s_C_	0.04 β	-	-	-
P_NC*_	−0.06	+0.20	−0.09	−0.16
P_NC_	+0.27	+0.20	+0.30	+0.27
P_HN_	-	-	-	0.0
P_HC*_	-	-	-	0.30

**Table 2:** B3LYP/6-21G band gap energies E_g_ and E_Γ_, ZPE, ^n^E_g_ and F_g_ for diamond.

Energy (eV)	Energy (eV)	Observed (eV)
E_g_	5.76	5.80
E_Γ_ (Γ_25′_–Γ_15_)	7.00	~7–7.4
ZPE	0.37	0.37
^n^E_g_	5.39	5.48

**Table 3:** B3LYP/6-21G spin-allowed (Δs_z_ = 0) and spin-forbidden (Δs_z_ ǂ 0) virtual transition energies in N_s_^0^ taken from Figure 2a, where the defect (N/C*) band width is 0.53 eV and the gap to the valence band upper edge, 1.9 eV.

Transition	Type	(eV)
α → α	indirect	3.02
α → α	direct	3.55
β → β	direct	4.60
α → β	indirect	2.22
α → β	direct	2.75
β → α	indirect	5.40
β → α	direct	5.47

**Table 4:** B3LYP/6-21G band gaps in N_s_^+^, N_s_^−^ and N_s_-H taken from Figure 2b–d where the spin-allowed and spin-forbidden transitions have identical energies.

	N_s_^+^		N_s_^−^		N_s_-H	
Gap	Type	(eV)	Type	(eV)	Type	(eV)
E_Γ_	direct	5.09	direct	2.55	direct	5.56
E_g_			indirect	1.81	indirect	4.86
					indirect	4.99
					indirect	5.43

**Table 5:** N_s_^0^ B3LYP/6-21G Γ-point gap (E_Γ_), direct SCF (Δ_SCF_) and absorption edge (E_g_) energies (eV) and changes to atomic charges (ǀeǀ), δq_N_, δq_C*_, δq_C_, atomic spins (ǀeǀ), δs_N_, δs_C*_, δs_C_, in N_s_^0^. t denotes a thermally excited state. α and β refer to changes in spin-up and spin-down orbitals charges and spins, respectively.

Transition	Type	E_Γ_	Δ_SCF_	E_g_	δq_N_	δq_C_*	δ_C_	δs_N_	δs_C_*	δs_C_
Donor band										
α → β	N(2s)C*(2p_y_,3p_y_)	2.75	1.96	1.43	+0.27	−0.16	−0.08	−0.09 ^α^	1.86 ^β^	0.04 ^β^
	N(2s)C*(2p_y_,3p_y_)t		1.88	1.35	*+0.01*	*+0.02*	*0.0*	−*0.01 ^α^*	*0.01 ^β^*	*0.0*
Valence bands										
α → α	N(3s,3p_y_)	3.62	4.23	3.67	−0.78	+0.11	+0.25	1.01 ^β^	0.08 ^α^	0.28 ^α^
β → β	N(3s,2p_y_)	4.60	4.72	-	−0.55	+0.12	+0.18	1.46 ^α^	1.14 ^α^	0.27 ^β^
	N(3s,3p_y_)		3.04	-	−0.80	+0.27	+0.24	1.48 ^α^	1.53 ^α^	0.30 ^β^
α → β	N(3s,2p_y_)	2.75	4.72	4.15	−0.55	+0.12	+0.18	1.46 ^β^	1.14 ^β^	0.27 ^α^
	N(3s,3p_y_)		3.04	2.46	−0.80	+0.27	+0.24	1.48 ^β^	1.53 ^β^	0.30 ^α^
	C*(3s,2p_y_)		1.00 ^#^	-	−0.01	−0.31	0.0	0.32 ^β^	2.00 ^β^	0.02 ^α^
	C*(2s,2p_y_,3p_y_)		1.54	-	+0.04	+0.07	0.0	0.25 ^β^	1.67 ^β^	0.01 ^α^

# charge transfer to the 3 C atoms nn to C*.

**Table 6:** N_s_^+^ B3LYP/6-21G Γ-point gap (E_g_/E_Γ_) and direct SCF energy (Δ_SCF_) (eV) and changes to atomic charges (ǀeǀ), δq_N_, δq_C*_, δq_C_, atomic spins (ǀeǀ), δs_N_, δs_C*_, δs_C_, in N_s_^+^. α and β refer to changes in spin-up and spin-down orbitals charges and spins, respectively.

Transition	E_g_/Γ	Δ_SCF_	δq_N_	δq_C_*	δ_C_	δs_N_	δs_C*_	δs_C_
N(3s,2p_y_)	5.09	5.16	–0.44	+0.13	+0.14	0.97 ^β^	0.40 ^α^	0.21 ^α^
N(3s,3p_y_)		2.34	–0.77	+0.33	+0.20	1.00 ^β^	0.41 ^α^	0.23 ^α^
C*(2s,3s,2p_y_,3p_y_)		4.66	+0.17	–0.65	–0.01	0.44 ^α^	1.29 ^β^	0.02 ^β^
C*(2s,3s,2p_y_)		4.17 ^#^	+0.01	–0.38	–0.01	0.21 ^α^	0.94 ^β^	0.02 ^β^
C*(3s,2p_y_,3p_y_)		2.51	+0.16	–0.53	–0.01	0.38 ^α^	0.93 ^β^	0.02 ^β^

# charge transfer to the 3 C atoms nn to C*.

**Table 7:** N_s_^−^ B3LYP/6-21G Γ-point gap (E_g_/E_Γ_) and direct SCF energy (Δ_SCF_) (eV) and changes to atomic charges (ǀeǀ), δq_N_, δq_C*_, δq_C_, atomic spins (ǀeǀ), δs_N_, δs_C*_, δs_C_, in N_s_^−^. α and β refer to changes in spin-up and spin-down orbitals charges and spins, respectively.

Transition	E_g_/Γ	Δ_SCF_	E_g_	δq_N_	δq_C*_	δ_C_	δs_N_	δs_C*_	δs_C_
N(3s,3p_y_)	2.55	3.94	3.36	–0.77	0.09	0.25	1.03 ^β^	0.01 ^α^	0.28 ^α^
C*(3s,2p_y_,3p_y_)		3.13 ^#^	2.55	0.05	–0.68	0.01	0.0	1.14 ^β^	0.01 ^α^
C*(2s,3s,2p_y_)		4.34 ^C^		-	-	-	-	-	-
C*(2s,2p_y_,3p_y_)		3.28 ^C^		-	-	-	-	-	-

C—conducting state; # charge transfer to the 3 C atoms nn to C*.

**Table 8:** N_s_-H B3LYP/6-21G 21G Γ-point gap (E_g_/E_Γ_) and direct SCF energy (Δ_SCF_) (eV), and changes to atomic charges (ǀeǀ), δq_H_, δq_C*_, δq_C_, atomic spins (ǀeǀ), δs_H_, δs_C*_, δs_C_, in N_s_-H. α and β refer to changes in spin-up and spin-down orbitals charges and spins, respectively.

Transition	E_Γ_	Δ_SCF_	E_g_	δq_N_	δq_C*_	δq_H_	δs_H_	δs_C*_	δs_H_
C*(2p_y_,3s,3p_y_)	5.56	4.32 ^#^	3.75	–0.03	–0.70	0.44	0.01 ^α^	0.40 ^β^	0.57 ^α^

# charge transfer to the 3 C atoms nn to C*.

## Data Availability

The data presented in this study are available on request from the corresponding author. The data are not publicly available due to ongoing research.
